# A Treatise on Fractures in the Vicinity of Joints, and on Certain Forms of Accidental and Congenital Dislocations

**Published:** 1847-07-01

**Authors:** 


### A Treatise on Fractures in the Vicinity of Joints, and on certain Forms of Accidental and Congenital Dislocations. By *Robert William Smith*, M.D., M.R.I.A., Fellow of the Royal College of Surgeons in Ireland, Lecturer on Surgery at the Richmond Hospital School of Medicine, &c. &c. Octavo, pp. 314. Dublin: Hodges and Smith, 1847.

We know of no class of cases more perplexing to the practitioner than the injuries considered in the work before us. Often have we seen surgeons even of considerable experience at fault, or at a loss to make out the exact nature of the lesion in cases of injury connected with a joint, and though much has been written on the subject, there is no work in which it has been satisfactorily treated of. Boyer, Sir A. Cooper, and Dupuytren have indeed done a good deal in clearing away the obscurities connected with fractures in the vicinity of the articulations, but there is a remarkable discrepancy in their accounts of the symptoms of many of these injuries, which can only be accounted for by unsettled and imperfect views of their pathological characters. We think, therefore, that Mr. Smith, who is known, by his contributions to the Dublin Medical Journal, to have devoted much attention to this important subject, required no apology for submitting his views to the notice of the profession.

Chapter I. is on the Diagnosis and Pathology of Fractures of the Neck of the Femur. After describing the ordinary symptoms of this injury, Mr. Smith notices the difference of opinion which has existed, and indeed still continues, as to the amount of shortening of the limb which occurs in the two varieties of this important lesion. "Upon this question the most eminent surgeons of modern times are directly opposed to one another. Sir Astley Cooper, Amesbury, Chassaignàc, Vidal (De Cassis), and others, maintain that the shortening is greater in the intracapsular fracture than in the extracapsular. Upon the other hand, Desault, Boyer, Dupuytren, Cloquet, Earle, Stanley, &c., have stated, that the greatest amount of shortening accompanies the fracture external to the capsule. How are such conflicting statements to be reconciled?" In discussing this question our author very properly limits his observations to the retraction of the limb, which *immediately* succeeds the receipt of the injury; for this is the period at which it is most important to form a correct diagnosis. The chief circumstance, according to which the degree of shortening varies in cases of intra-capsular fractures, is, the amount of laceration suffered by the fibrous covering of the neck of the bone, which Mr. Smith rather inappropriately terms the "cervical ligament of the femur."

"If the force which acts upon the neck of the femur be inconsiderable, and, as it were, exhausted, after producing the fracture, this ligament may escape uninjured, or nearly so. In such a case, the retraction of the limb will be very slight, and will be at its minimum when the fracture has traversed the bone obliquely from the inferior part of the head of the femur downwards and outwards. When the cervical ligament remains nearly entire, it not only limits the retraction of the limb, but it likewise becomes the sole medium through which a vascular communication is maintained between the fragments; it is, therefore, very important to observe the utmost caution in examining the limb; and if the existence of fracture of the neck of the femur is rendered evident by the presence of the other symptoms which characterize this lesion, we should, I think, abstain from instituting such an examination as is necessary to produce crepitus. In doubtful and obscure cases, this examination should of course be cautiously made, but I perfectly coincide with the opinion of Boyer, that, in the majority of cases of fracture of the neck of the femur within the capsule, the nature of the injury is sufficiently indicated by other symptoms." P. 8.

No doubt, as stated by Boyer, and indicated in a case of Mr. Stanley's, which is quoted by the author, much injury may result from a rough examination of the injured limb, but without either shortening or eversion, which may not be present when the fibrous covering is entire, the nature of the case would not be so clear as to justify the surgeon in dispensing with an examination of the limb.

Among the causes which limit the retraction of the limb in cases of intra-capsular fractures, Mr. Smith does not omit to notice the powerful capsular ligament which so closely embraces the articulation. He very properly controverts the statement of Sir A. Cooper, that when the bone is broken within the capsule, the leg becomes from one to two inches shorter than the other, siding on this point with Mr. Earle and Boyer, who never witnessed such an occurrence. But when the fracture is external to the capsule and not impacted, there is but little to prevent the full force of muscular action upon the lower fragment of the bone, while, at the same time, the upper is depressed by the weight of the body, so that, from these two causes, a degree of shortening may be produced equal to, or even greater, than the entire length of the neck of the bone.

" Again, we meet with cases in which the shortening of the limb is not by any means decided or evident for several days after the receipt of the injury: in such instances it usually happens that the muscles have been, to a certain extent, paralyzed by contusion; but according as, under the influence of rest, &c., they regain their power of contracting, the limb becomes slowly and gradually shortened, and this independent of any process of absorption. A third class of cases occur, in which the retraction of the limb, having been at first scarcely perceptible, at the expiration of a few weeks becomes very considerable, owing to the rapid absorption of the neck of the bone. In the case of Margaret Myler the amount of shortening was at first only a quarter of an inch, but at the end of six weeks amounted to one inch and a half. Lastly, examples are sometimes seen in which the limb retains nearly its natural length for many weeks after the receipt of the injury, and then a very decided and, comparatively speaking, considerable degree of shortening occurs, not gradually, but suddenly. In these cases the diagnosis is somewhat obscure; the cause which has produced the fracture is comparatively slight, and the patient has not made any attempt to use the limb after the receipt of the injury; the eversion of the foot is by no means so well marked as when the retraction has occurred early; the patient is, it is true, unable to raise the limb *en masse,* but this may be owing to the effects of contusion upon the muscles; in fact, in cases such as those to which I allude, there may be at first neither shortening of the limb to any appreciable extent, eversion of the foot, nor any change in the position of the trochanter; and therefore, unless we can ascertain crepitus, it is most difficult to decide whether the neck of the bone be broken or not; and if we fail to elicit crepitus, the more prudent course is to withhold our opinion until time shall more fully develop the nature of the injury. In these cases the surgeon is apt to allow greater freedom to the patient than is advisable, or, it may be that, in his anxiety to establish the nature of the injury by producing crepitation, he employs too much force in rotating the femur; in either case it is highly probable that the sudden occurrence of shortening of the limb will establish the nature of the lesion which the joint has suffered; the eversion of the foot now becomes decided, and the trochanter altered in position. These phenomena may take place long after the occurrence of the accident. In these instances it is most probable that, at the time of the receipt of the injury, the cervical ligament, having escaped laceration, prevented the retraction of the limb, but that subsequently it was torn, either in consequence of some imprudent exertion upon the part of the patient, or too eager a desire upon that of the surgeon to produce crepitus by forcible extension and rotation of the limb; the retraction then takes place as the immediate result of the laceration of this important membrane." P. 12.

Mr. Smith states, that if there be any one fact in surgical pathology more certain than another, it is this—that in cases of fracture of the neck of the femur within the capsular ligament, there is scarcely any callus ever effused; it certainly is never formed in such quantity as to be at all capable of counteracting the causes which produce shortening of the limb. We shall not follow our author in his rather tedious refutation of the views of M. Rodet on the subject of shortening after fractures, to which undue attention seems to have been bestowed by him. The position of the foot is not subject to as much variety as the shortening of the limb; in general it is turned outwards. Mr. Smith's experience would lead him to say that inversion of the foot is most frequently seen in cases of extra-capsular fractures. He has seen several examples in which the foot was turned inwards, in five of which the fracture was external to the capsule. He remarks—

"There is one remarkable circumstance which appears to have escaped the observation of those who have described injuries such as those now alluded to, accompanied by inversion of the foot, and which appears to support the opinion that this symptom is to be ascribed to the relative position of the fragments, rather than to the influence of muscular action. I have observed it several times, and it is this: the deformity having been removed, and the limb restored to its natural length by extension, as soon as the extending force ceases to act, though the limb is again shortened, the foot will be found to remain everted." P. 25.

In every case of fracture of the neck of the femur, accompanied by inversion of the foot, which Mr. Smith has had an opportunity of examining after death, the inferior has been placed in front of the superior fragment. He gives the best description that we have hitherto met with of that remarkable but not uncommon species of fracture of the neck of the femur, termed the impacted fracture, in which the broken cervix is driven into the shaft of the femur between the trochanters. Our author justly observes:—

"This peculiar form of fracture is the only one, the diagnosis of which is attended with difficulty, for it rarely happens that the limb is shortened to the same extent as in the ordinary examples of extracapsular fractures; in general the amount of retraction is nearly the same as in cases of fracture within the capsule. Between these two forms of injury, therefore, the shortening of the limb is not available as a means of differential diagnosis; upon further examination, however, it will generally be found that crepitus cannot be elicited upon rotating the limb, because upon the one part the neck of the femur is so firmly wedged into the cancellated tissue of the shaft, that the fractured surfaces cannot be moved upon each other; and upon the other the integrity of the strong fibrous and tendinous structure which invests the whole of the region traversed by the second fracture, which has been already alluded to as being always present, is such that the detached portion of the trochanter, whether large or otherwise, in general moves with the shaft of the bone, and it is only by submitting the patient to an examination unjustifiably severe, that we are occasionally enabled to produce this characteristic evidence of fracture. When we endeavour to extend the limb, we usually find that no force which it is safe to employ will restore it to its normal length, and in general the dislocated head of the femur will return into its socket more readily than the impacted cervix will leave the cavity between the trochanters, into which it has been driven.

" More remarkable deviations than these, from the ordinary symptoms of fracture of the neck of the femur, sometimes attend this particular lesion; and cases have occurred in which the patient has not only raised himself from the ground after the fall which caused the fracture, but has even walked a considerable distance, bearing his weight upon the injured limb." P. 29.

These cases are very liable to be mistaken for contusion of the hip. The evidence of the existence of an impacted fracture of the cervix femoris is of a negative rather than of a positive character, and is thus briefly stated. " 1. Slight shortening of the limb. 2. Slight eversion of the foot. 3. Absence of crepitus. 4. Great difficulty in all cases, and in the majority of instances an impossibility, of removing the shortening of the limb by extension; and lastly, less loss of power than in other forms of fracture of the neck of the femur." The anatomical characters of this form of fracture, as well as of the fracture of the neck with the capsular ligament, are well described by Mr. Smith, and obviously from extensive observation of specimens of those injuries.

Our readers are probably aware that a peculiar form of injury of the neck of the femur, termed "partial fracture," has been described by the late Mr. Colles and also by Mr. Adams of Dublin. The former author has spoken of its occurrence in cases of fracture within the capsular ligament, and the latter has described the symptoms and the morbid appearances supposed to characterize it in extra-capsular fractures. Mr. Smith, after quoting the observations of Mr. Adams on this form of fracture, states that he has examined the specimens referred to by both these pathologists, and for reasons which are alleged he can come to no other conclusion than that the doctrine of partial fracture has not yet been proved to be correct.

In reference to that long-agitated question, the possibility of osseous union taking place in cases of fracture of the neck of the femur within the capsular ligament, Mr. Smith, after briefly stating and repeating the chief objections which have been made to the occurrence of such an event, gives a concise summary of the cases which constitute the evidence by which the possibility of osseous union taking place has in his opinion been established. Seven cases, most of them well known to surgical pathologists, are adduced. Our author thinks it highly probable that they have all been examples of impacted fractures: " certainly in all those, of which delineations have been given, there has been either penetration of one fragment by a portion of the other, or else the irregularity of the line of fracture has been such that the displacement of the fragments has been prevented; they have been maintained in contact and at rest, and it is under such circumstances alone that we are to hope for the occurrence of bony consolidation."

Mr. Smith, after noticing the unfavourable nature of the prognosis in cases of fracture of the neck of the femur, the injury in many instances soon proving fatal, and in all the functions of the limb being for ever impaired, makes a remark which we can fully confirm, viz., that the form of fracture which is most rapidly and most frequently fatal is the extra-capsular fracture, when it is accompanied by a comminuted fracture with displacement of the trochanters. Our author next gives a brief account of the preparations which seem to warrant the conclusions he has deduced from them, accompanying the description with a concise history of the symptoms which each case presented during life. The preparations amount to sixty in number, and are nearly all illustrated by woodcuts. He subjoins a table shewing the age of the patient, the degree of shortening of the limb, and the position of the foot in these cases, as well as the length of time which elapsed between the receipt of the injury and the death of the patient. He terminates this elaborate investigation of fractures of the neck of the femur with the following conclusions:—

"1. A slight degree of shortening, removable by a moderate extension of the limb, indicates a fracture within the capsule.

"2. The amount of *immediate* shortening, when the fracture is within the capsule, varies from a quarter of an inch to one inch.

"3. The degree of shortening, when the fracture is within the capsule, varies chiefly according to the extent of laceration of the cervical ligament.

"4. It also varies according as the fracture is impacted or otherwise.

"5. In some cases of intracapsular fractures, the injury is not immediately followed by shortening of the limb.

"6. This is generally to be ascribed to the integrity of the cervical ligament.

"7. In such cases, shortening may occur suddenly, at a period more or less remote from the receipt of the injury.

"8. This sudden shortening of the limb is in general to be ascribed to the accidental laceration of the cervical ligament, previously entire, and is indicative of a fracture within the capsule.

"9. The deposition of callus around the fragments is not necessary for the union of the intracapsular fracture.

"10. When osseous consolidation occurs in the intra-capsular fracture, it is effected by the direct union of the broken surfaces, which are confronted to each other.

"11. The osseous union of the intracapsular fracture is most likely to occur when the fracture is of the variety termed 'impacted.'

"12. In the intracapsular fracture, the mode of impaction is different from that which obtains in the extracapsular.

"13. The degree of shortening, when the fracture is external to the capsule, and does not remain impacted, varies from one inch to two inches and a half.

"14. When a degree of shortening occurs immediately after the receipt of the injury, we usually find a comminuted fracture external to the capsule.

"15. The intracapsular fracture is accompanied by fracture with displacement of one or both trochanters.

"16. The extracapsular *impacted* fracture is accompanied by fracture without displacement of one or both trochanters.

"17. In such cases, the fracture of the trochanters unites more readily than that of the neck of the bone.

"18. The degree of shortening, in the intracapsular impacted fracture, varies from a quarter of an inch to an inch and a half.

"19. The exuberant growths of bone met with in these cases have been erroneously considered to be merely for the purpose of supporting the acetabulum and the neck of the femur.

"20. The final cause of their formation is the union of the fracture through the posterior intertrochanteric space.

"21. The difficulty of producing crepitus, and of restoring the limb to its normal length, are the chief diagnostic signs of the impacted fracture.

"22. The position of the foot is influenced principally by the obliquity of the fracture, and the relative position of the fragments.

"23. Inversion of the foot may occur in any of the varieties of fracture of the neck of the femur.

"24. When the foot is inverted, we usually find that either a portion or the entire of the extremity of the lower is placed in front of the superior fragment.

"25. In cases of comminuted extracapsular fractures, with fracture and displacement of the trochanters, the foot will generally remain in whatever position it has been accidentally placed: it may be turned either inwards or outwards, or there may be inversion at one time and eversion at another.

"26. Severe contusion of the hip-joint, causing paralysis of the muscles which surround the articulation, is liable to be confounded with fracture of the neck of the femur.

"27. Severe contusion of the hip-joint may be followed, at a remote period, by shortening of the limb and eversion of the foot.

"28. The presence of chronic rheumatic arthritis may not only lead us to suppose that a fracture exists when the bone is entire, but also, when there is no doubt as to the existence of fracture, may render the diagnosis difficult, as to the seat of the injury with respect to the capsule.

"29. Severe contusion of the hip-joint, previously the seat of chronic rheumatic arthritis, and the impacted fracture of the neck of the femur, are the two cases most likely to be confounded with each other.

"30. Each particular symptom of fracture of the neck of the femur, separately considered, must be looked upon as equivocal; the union of all can alone lead to the formation of a correct opinion as to the nature and seat of the injury." P. 112.

In Chapter II. there is an account of the disease of the hip-joint termed by Mr. Adams "Chronic Rheumatic Arthritis." The symptoms of the disease, as described by Mr. Smith, are those of a chronic rheumatic affection with which we have long been familiar, though we have not hitherto connected them with the remarkable changes in the neck and head of the femur described and represented in the work before us. Since our attention has been called to these changes by the interesting observations of Mr. Adams in the Cyclopædia of Anatomy, we have been led to question whether the shortening of the neck, and the curious alteration in the form and depression of the head, occasionally met with, are altogether dependent upon a peculiar morbid change. We are satisfied that they may take place in advanced life as the result of the degeneration to which the bones are liable, without being attended with any rheumatic affection or marked symptom of disease. In this natural decay, the animal parts sometimes waste faster than the earthy, and thus the bone becomes so brittle that it is liable to fracture from the slightest violence; whilst in other cases the earthy parts are removed before the animal, so that the bone, being softened, becomes depressed and changed in shape by the pressure to which it is subject. This latter change may, and indeed is very liable to be precipitated in chronic rheumatism of the joint,—the state of rest consequent on the painful nature of the affection, being favourable to the natural process of degeneration. The exuberant growths of bone, and eburnation of the bared head of the femur, combined with the other changes, are very probably the result of chronic rheumatism. There are some excellent woodcuts, illustrating the various alterations in the femur described in this chapter. Our own experience leads us to coincide with Mr. Smith in the following practical remarks :—

"It is an affection amenable to treatment in a very slight degree, and although its anatomical characters would lead us to suppose that it depended upon chronic inflammation affecting all the tissues entering into the composition of the joint, yet it is not found that antiphlogistic treatment produces any material alleviation of pain, nor is any permanent benefit derived from local bleeding or counter-irritation. Patients labouring under this affection not unfrequently present themselves at hospitals and dispensaries, in whom the entire of the region of the hip is covered with the marks of leeches, cupping, moxa, &c., but the disease has, notwithstanding, steadily progressed, totally uninfluenced by such treatment. Rest, anodyne embrocations, keeping the joint protected by new flannel or carded wool from the influence of cold and damp, together with the free and long-continued use of hydriodate of potass, combined with the compound decoction of sarsaparilla, and small doses of colchicum, constitute the mode of treatment from which I have seen most benefit derived." P. 128.

The subject of Chapter III. is Fractures of the Bones of the Fore-arm in the vicinity of the Wrist-joint. In by far the most common form of fracture in this situation the lower end of the radius is broken and its lower fragment displaced backwards. This is an obscure form of injury until recently not well understood, to which the attention of the profession was called by an accurate description of it, published by Mr. Colles, in 1814, in the 10th volume of the Edinburgh Med. and Surg. Journal.* It has been particularly noticed by Dupuytren, and is also described by Chelius. Mr. Smith, after quoting Mr. Colles' account of this injury, gives some minute particulars of the symptoms which characterize it. The marked deformity at the wrist is well shown in the accompanying woodcuts. It has been supposed, from the symptoms of the injury, that the fracture is in many instances oblique. This is not the opinion of Mr. Smith. He states :

"I have lately examined upwards of twenty specimens of the injury, and have not found one in which the bone had been broken with any considerable degree of obliquity from above, either downwards and backwards, or downwards and forwards ; in all of them the anterior and posterior margins of the fractured surface have been nearly upon the same level and the surface plane ; there is, however, an obliquity which the fracture not unfrequently presents, and which is directed either from

---

* Mr. Smith finds fault with the learned author of the Surgical Dictionary for not alluding to Mr. Colles' account of this fracture, remarking that it is the duty of every person who undertakes to write upon a given subject, to make himself, as far as possible, acquainted with, and also to acknowledge, the labours of those who have preceded him in the same field of enquiry. The observation is a just one, but it is one, we think, particularly applicable to many of the writers of the Dublin School, though not to Mr. Smith.

within downwards and outwards, or from without downwards and inwards ; even this obliquity, however, is always trifling, and scarcely ever sufficiently great to invalidate the truth of the general proposition (first maintained, I believe, by Monsier Voillemier), that when the radius is broken within an inch of its lower extremity, the direction of the fracture is transverse, the direction which we would expect it to follow, from reflecting upon the manner in which the accident happens, and in which the force which breaks the bone is applied; for, when the fracture takes place from a fall upon the palm of the hand, as it usually does, the force, though principally and ultimately transmitted to the posterior surface of the lower end of the radius, yet is applied in a direction nearly parallel to the plane of the carpal surface of the bone." P. 142.

It appears that Voillemier, a writer in the Archives Génerales de Medicine, regards all fractures of the lower end of the radius as examples of impacted fracture, and his views are given in the work before us at some length, and afterwards very fully discussed. This opinion is founded upon the fact that, in nearly all specimens of fracture of the lower end of the radius examined long after the occurrence of the injury, a line of compact tissue is found, continuous with the posterior wall of the shaft, and extending to a greater or less distance into the reticular structure of the lower fragment. Mr. Smith's attention having been directed to the subject by the memoir above referred to, he examined numerous sections of this fracture, and, for reasons which we have not sufficient space to quote, he considers that the doctrine " of fracture with penetration" is untenable. He believes that " the impaction is only apparent, and that the compact tissue of the shaft is not found enveloped in bone, from its having penetrated the lower fragment at the time of the occurrence of the injury, but because it becomes subsequently incased in osseous matter during the process by which the bony union of the fracture is accomplished." We are disposed to agree with Mr. Smith on this subject, but we think that he has discussed the point at greater length than its importance would seem to require.

Mr. Smith describes a very rare injury, viz. " fracture of the lower extremity of the radius, with displacement of the lower fragment forwards." It generally occurs in consequence of a fall upon the back of the hand.

" It is accompanied by great deformity, the principal features of which are a dorsal and a palmar tumour, and a striking projection of the head of the ulna at the posterior and inner part of the fore-arm; the dorsal tumour occupies the entire breadth of the fore-arm, but is most conspicuous internally, where it is constituted by the lower extremity of the ulna displaced backwards; from this point, the inferior outline of the tumour passes obliquely upwards and outwards, corresponding in the latter direction to the lower end of the superior fragment of the radius. Immediately below the dorsal swelling there is a well-marked sulcus, deepest internally below the head of the ulna, directed nearly transversely, but ascending a little as it approaches the radial border of the fore-arm.

" The palmar is less remarkable than the dorsal tumour; formed principally by the lower fragment of the radius, it is obscured by the thick mass of flexor tendons which cross the front of the carpus, but towards the ulnar border of the limb there is a considerable projection, which marks the situation of the pisiform bone, passing down to its attachment into which, can be seen the tendon of the flexor carpi ulnaris thrown forwards in strong relief. The transverse diameter of the fore-arm is not much altered, but the antero-posterior is considerably increased, and the radial border of the limb becomes concave at its lower part." P. 163.

Mr. Smith has never had an opportunity of ascertaining the anatomical characters of this fracture. He is quite satisfied, however, of the nature of the injury, and believes that it has not unfrequently been mistaken for dislocation of the carpus forwards.

"The facility, however, with which the deformity can be removed, its liability to recur when the extending force ceases to act, the production of crepitus when the limb is extended, and a motion of rotation given to the hand, and our being able to feel the irregular margin of the upper fragment of the radius posteriorly, are sufficient to enable us to distinguish this accident from luxation of the bones of the wrist forwards." 163.

Another form of injury occurring in the vicinity of the wrist is "a separation of the inferior epiphysis of the radius, with fracture of the lower extremity of the ulna." This accident is very liable to be mistaken for dislocation of the wrist backwards, but cannot be confounded with the ordinary fracture of the radius; the dorsal tumour is transverse, and the limb presents none of the peculiar deformity arising from the displacement of the lower fragment of the radius towards the side of supination which distinguishes the more common form of fracture.

"Although this injury assumes very much the appearance of dislocation of the carpus backwards, it may yet be distinguished from it without any considerable difficulty, more especially should we be fortunate enough to see it, before the occurrence of tumefaction has obscured the diagnostic signs. The styloid processes of the radius and ulna can be felt, still holding their normal relations to the carpus, and, as has been remarked by Boyer, these processes move with the hand when any motion is imparted to the latter. If the distance between the superior margin of the dorsal tumour, and the extremity of the middle finger of the injured limb, be measured and compared with that between the corresponding point of the hand and the upper edge of the carpus of the sound limb, the former measurement will be found to exceed the latter by at least half-an-inch; sometimes the difference is greater; but if the case should be one of dislocation of the wrist backwards, this measurement will give the same results upon each side, and the styloid processes will be found to remain at rest when the hand is moved; if we add crepitation, the easy reduction of the deformity by extension, and its liability to recur when the extending power is removed, we are at once furnished with a group of symptoms, quite sufficient to enable us to distinguish this injury from that very rare accident, luxation of the wrist." P. 166.

With respect to the treatment of the ordinary fracture of the lower end of the radius with displacement of the lower fragment backwards, the object most difficult to be accomplished is to restore to the carpal surface of the bone its normal direction forwards, and this is not effected by the means recommended by Dupuytren. Mr. Smith has found the following mode of treatment to answer remarkably well, and to fulfil all the more important indications.

"The deformity having been, as far as possible, removed by extension and counter-extension, and the hand moderately adducted, a cushion is to be placed upon the posterior surface of the limb, of sufficient length to extend from the elbow to the fingers; the portion of this cushion which corresponds to the lower fragment of the radius and to the carpus should be thicker than any other part, and from its ulnar to its radial border, should gradually increase in thickness. A transverse section of this portion of the cushion would represent an isosceles triangle, the base of which would correspond to the radial border of the limb.

The objects proposed to be attained by constructing the pad of this form are, to press the lower fragment of the radius forwards, and to direct its external border towards the side of pronation.

" A second cushion, thicker below than above, is to be placed upon the front of the limb, but should not descend below the margin of the superior fragment, for otherwise it would, to a certain extent, counteract the influence of the dorsal cushion, and would tend to maintain the displacement backwards of the inferior fragment. An anterior and a posterior splint are then applied, each of which should be at least an inch broader than the fore-arm; the posterior should extend from the elbow to the fingers, and should be curved from the wrist downwards, to receive the adducted hand; the anterior need not descend below the palm of the hand: a roller is then to be carried around the splints in the ordinary manner.

" By constructing and placing the cushions in the manner above described, the two fragments are pressed in opposite directions, and the carpal surface of the inferior is directed forwards, while the curved splint, by maintaining the hand moderately adducted, tends to restore to the articulating surface its natural direction inwards, quite as effectually as the ulnar splint of Dupuytren, and with much less uneasiness to the patient. It is scarcely necessary to mention, that the form which I have recommended can be readily given to that portion of the dorsal cushion which corresponds to the lower fragment, by the employment of graduated compresses." P. 168.

" During the application of the apparatus, as well as during the subsequent treatment, the fore-arm should be maintained in a position intermediate between pronation and supination, for it is in this position that the co-aptation of the fragments can be most easily and most perfectly accomplished, in consequence of the relaxation of the pronator and supinator muscles." P. 169.

We may here add the description of the plan of treatment in these cases recommended by Professor Fenger of Copenhagen, and recently communicated to the Royal Medical and Chirurgical Society.* He states that, as the deviation occurs in a curve, with its centre upon the fracture, it is desirable to counteract the deformity by extension acting in a direction according to the tangent of that curve. This end he thinks is best attained by acting through the medium of the hand and of the capsular ligament which is attached to the lower end of the radius. The hand is first to be brought into a position of strong flexion, and the fore-arm is then placed on an oblique plane, with the carpus highest, the hand being permitted to hang freely down the perpendicular end of the plane. The tendons of the extensor muscles are thus brought into a position which enables them to assist in keeping the reduced fragments of the bone in proper relation. Where the deformity requires it, the displaced lower fragment is to be pressed into its position by the thumb of the operator, after sufficient extension has been made, and when the hand is bent on the fore-arm. The patient is to be kept in bed, but the hand is not confined, the seat of fracture being covered only by an evaporating lotion.

Mr. Smith's views are summed up in a number of general corollaries, which we regret that we are unable to find room for. This chapter presents the clearest view of the anatomical characters, symptoms, and diagnosis of the common fracture of the lower end of the radius that we know

---

* The Lancet, Vol I., 1847, p. 487.

of, and its obscure nature and the difficulty of managing it, perhaps justify the length with which it is treated.

Chapter IV. treats of "Fractures of the Humerus, in the vicinity of the Shoulder-joint." On the subject of Fractures of the greater tuberosity of the humerus we find nothing particularly deserving for notice. Mr. Smith has had but one opportunity of observing the anatomical characters of the injury. The accident had occurred many years before death, and the history connected with it could not be precisely ascertained.

Our author describes two varieties of impacted fracture of the neck of the humerus. In one, the upper extremity of the lower fragment penetrates the reticular tissue of the head of the bone; "this is an extracapsular fracture, and occupies the situation which, in the young subject, marks the junction of the epiphysis with the shaft; in the other, the superior fragment is forced downwards into the cancellated structure between the tubercles, the greater of which processes is, in almost every such instance, split off from the shaft of the humerus; in this case the fracture is intracapsular, and occurs through the anatomical neck of the bone." Both these injuries are fractures of the true anatomical neck of the humerus, which, our experience would lead us to say, are of very rare occurrence. They have been confounded with fractures through the tuberosities, or through the line of junction of the epiphysis with the shaft of the bone.

"In the latter, the deformity is considerable, in consequence of the lower fragment being drawn inwards by the muscles which constitute the folds of the axilla; but in the former there is scarcely any displacement of the inferior fragment, the influence of these muscles being counteracted by the supra-spinatus, infra-spinatus, and teres minor, attached to the greater tubercle; the bone being thus placed between two opposing forces, suffers very little displacement, and the deformity is slight in proportion; hence the diagnosis of fracture of the anatomical neck of the humerus is, comparatively speaking, obscure. The impairment of the motions of the joint and crepitus are, in fact, almost the only symptoms upon which we can depend, in forming our opinion as to the nature of the injury which the bone has sustained." P. 185.

Notwithstanding the unfavourable circumstances in which the head of the humerus is placed as regards bony union, osseous consolidation takes place, the impaction serving to maintain the parts in contact. Mr. Smith describes some interesting cases and specimens of the injuries in question.

"Separation of the superior epiphysis from the shaft of the humerus," is an accident which Mr. Smith states not unfrequently occurs in early life. Our experience would lead us to say that it was a rare form of injury. It is attended by a considerable degree of deformity, but of so striking a character, that there is no great difficulty in recognising the true nature of the injury.

"The axis of the arm is directed from above, within, and before, downwards, outwards, and backwards; the elbow, however, projects but little from the side, and can be brought into contact with it with facility; the head of the bone can be distinctly felt in the glenoid cavity; a slight depression is seen beneath it, and it remains motionless, when the shaft of the humerus is rotated.

"The most remarkable feature, however, of this injury, is a striking and abrupt projection, situated beneath the coracoid process, and caused by the upper extremity of the lower fragment or shaft of the bone, drawn inwards by the muscles which constitute the folds of the axilla: there is but little displacement as regards the length of the bone, for the extremity of the inferior fragment is seldom drawn so far inwards as to enable it to clear completely the surface of the superior. Were this to occur, the humerus would, of course, be drawn upwards by the muscles passing from the shoulder to the arm, in a direction parallel, or nearly so, to the axis of the humerus, and a corresponding diminution in the length of the limb would result.

"This remarkable and abrupt projection does not present the sharp, irregular margin of an ordinary fracture; on the contrary, it feels rounded, and its superior surface is smooth and slightly convex. The latter can be felt as plainly as the cup-like cavity of the head of the radius, in cases of luxation of that bone backwards at the elbow-joint. By pressing the upper end of the lower fragment outwards, and directing the elbow inwards, during extension and counter-extension, crepitus can be perceived, and the deformity removed without much difficulty; but the moment the parts are abandoned to the uncontrolled action of the muscles, the deformity recurs." P. 201.

Fractures of the Acromial Extremity of the Clavicle are considered in Chapter V. The chief point of interest in relation to this inquiry adduced by our author, is the occurrence of displacement of the outer fragment of the clavicle in cases of fracture between the trapezoid ligament and the acromio-clavicular articulation, which, he says, is, in general, considerable, its inner extremity being drawn inwards. This displacement is frequently carried to such an extent that the fragments form a right angle with each other; and it is principally due to the action of the clavicular portion of the trapezius muscle. In consequence of the displacement the clavicle is shortened.

In Chapter VI. Mr. Smith treats of Dislocations of the Bones of the Foot. After briefly alluding to the displacements with which surgeons are already familiar, he remarks that the instances of luxation he is about to describe are different from any that have yet been recorded. They are instances of dislocation of the metatarsus and internal cuneiform bone, upwards and backwards upon the tarsus. Its external characters are said to be very striking, and clearly to indicate the nature of the accident.

"The foot is greatly deformed, but it is at first sight obvious, that the relations which the bones composing the ankle-joint bear to each other, are undisturbed. There is, it is true, a remarkable fore-shortening of the foot, but we are not likely, on this account, to confound the accident with displacement of the tibia forwards, for there is no corresponding elongation of the heel: the foot, in front of the ankle-joint, is shortened to the extent of an inch or more, but the heel preserves its natural relations to the bones of the leg.

"The foot likewise appears to be rotated upon its long axis, in such a manner that the aspect of the dorsal region is directed outwards, and that of the plantar inwards; the inner edge of the foot is elevated and the outer depressed. These alterations resemble those which are the results of the dislocation of the tibia outwards; but in the latter accident the outer edge of the foot is applied to the ground throughout its whole length, whereas in the injury under consideration, it is only the central third of the external margin of the foot, which is presented to the ground in walking.

"The next remarkable symptom which attracts observation, is the alteration which the form of the sole of the foot undergoes. Instead of presenting its natural concavity, the plantar region becomes convex, both in its antero-posterior and transverse diameters; and hence it is that, in standing or walking, the central third alone, or nearly so, of the outer margin of the foot, is presented to the ground, as has been already mentioned."

"The dorsal region of the foot presents a transverse prominence, situated about an inch below, and in front of the ankle-joint; commencing almost imperceptibly at the external margin of the foot, it gradually becomes more evident till it terminates in a very distinct projection at the inner edge of the tarsus. Between this prominence and the ankle-joint there is a superficial sulcus. The line of the transverse convexity in the plantar region, is opposite to the ridge upon the dorsum of the foot, but its most prominent part is at the outer edge of the tarsus." P. 226.

Such are the principal diagnostic signs of this dislocation. Mr. Smith has twice had an opportunity of ascertaining by *post-mortem* examination the precise nature of the accident, the luxation having remained unreduced in both instances. The anatomical characters presented in these cases are minutely described, but we must refer our readers to the work, in which they will also find several wood-cuts, without seeing which they would scarcely be able to comprehend Mr. Smith's description of this peculiar displacement of the tarsal bones.

"This remarkable and rare dislocation may be produced by the passage of a heavy body over the dorsum of the foot, but is much more liable to occur when a person, in falling or leaping from a considerable height, alights upon the anterior part of the foot. Under these circumstances, the limb is submitted to the operation of two forces acting in opposite directions; one, the weight of the body and impulse of the fall, tending to depress the tarsal bones, the other, the resistance of the ground, tending to displace the metatarsus upwards: the articulating surfaces thus glide past each other, and the anterior part of the foot is then drawn backwards, and the aspects of its surfaces altered by muscular action." P. 233.

In Chapter VII., on Congenital Luxations of the Wrist-joint, Mr. Smith quotes a case, recorded by Cruveilhier, as an example of luxation of the carpus forwards, but which so closely corresponds with the appearances in a case dissected by our author and regarded as a congenital luxation, that he is compelled to arrive at the conclusion that the French pathologist is in error, and that the lesion in his case is also an original malformation.

Chapter VIII., which treats of Congenital Dislocation of the shoulder, is an extension of a memoir published by Mr. Smith, in the 15th Volume of the Dublin Medical Journal.

Mr. Smith has ascertained the existence of two varieties of this malformation. "In one of these, the head of the humerus is placed beneath the coracoid process; while in the other and more rare variety, it is lodged in an abnormal socket, formed upon the dorsum of the scapula, below the outer and posterior part of the acromion. They may be termed the sub-coracoid and sub-acromial congenital luxations, and may exist either upon one side only, or upon both." Of the latter species our author has seen only one example, but several instances of the former have come under his observation within the last few years. Five cases are detailed, and representations given of certain alterations in the form of the head of the humerus and of the glenoid cavity, which were noticed in some of the cases. This is a lesion involved in some obscurity, and it is by no means clear that all the cases were really instances of congenital malformation.

Chapter IX. is on Dislocations of the Lower Jaw. Mr. Smith relates a very curious case of what he terms, we think incorrectly, congenital luxation of the inferior maxilla in a man, æt. 38, an idiot from his infancy, who died of diseased lungs.

"While engaged in examining the head, a singular deformity of the countenance, which is accurately represented in the accompanying figure, attracted my observation. The right and left sides of the face seemed as though they did not belong to the same individual, the left being in every respect larger and more fully developed. Upon this side the prominence of the malar bone and of the arch of the zygoma, the development of the masseter muscle, and the fulness of the parotid space, all in a remarkable manner contrasted with the atrophy of the opposite half of the countenance, which, in the situation of the zygoma, presented a concavity in place of a convexity, and in the parotid space a very distinct depression. The countenance was, moreover, crooked in almost every feature. Upon the right side the angle of the mouth was higher than upon the left, while upon the left the orbit was placed a little higher than upon the right; the superciliary arch was much more projecting, and the eye more prominent.

"The right side of the face appeared as it were sunk in, and the extremity of the finger could be placed between the parotideal margin of the jaw and the front of the external auditory canal."

"Upon elevating the integuments, and contrasting the dissected muscles of either side, those of the right were found to be much smaller than their fellows of the left side. The masseter, in particular, was atrophied both in length, breadth, and thickness; the temporal and pterygoid muscles also appeared small when compared with those of the opposite side, but as regarded colour and consistence, the muscular fibre did not deviate from the normal condition." P. 276.

"When the mouth was closed, and the teeth maintained in apposition, as exact as the abnormal state of the parts admitted of, the external lateral ligament of the lower jaw, instead of being directed backwards, was seen descending obliquely forwards, to be attached to a very imperfectly-developed condyle, which was not in contact with that portion of the temporal bone which, in the natural state, corresponds to the glenoid cavity, being separated from it by an interval of at least a quarter of an inch.

"There was neither an interarticular cartilage nor cartilage of incrustation, the osseous surfaces of the joint being invested by a thick periosteum alone. There was no distinct capsular ligament." P. 277.

"The right side of the inferior maxillary bone was considerably smaller than the left, the atrophy extending forwards to within a very short distance of the symphysis, and affecting the bone as to its length, breadth, and thickness, the ramus being half an inch less in its transverse diameter, and its parotideal margin half an inch shorter than upon the opposite side.

"The lower edge of the bone presented a deep concavity at its posterior part, and the angle was remarkably prominent and curved outwards; the parotideal margin, thin, concave at its upper part, and forming nearly a right angle with the body of the bone, terminated above in a small curved process, directed nearly horizontally inwards, its superior surface being directed slightly outwards, and its inferior slightly inwards.

"This process, which in form somewhat resembled the coracoid process of the scapula, was the only vestige of the existence of a condyle, and was destitute of cartilage.

"The external pterygoid muscle was attached to its anterior and inner part, and the external lateral ligament to its outer surface; there was a complete arrest of development in the condyle; the coronoid process was small and thin, and the sigmoid notch could scarcely be said to exist." P. 279.

The temporal malar, superior maxillary and right side of the sphenoid were also more or less atrophied. The right orbit was in consequence smaller than the left. The figure of the base of the skull shows also a diminution in the size of the arch formed by the occipital and parietal bones, though this is not mentioned by Mr. Smith. The case seems to us simply one of arrest of development of all the bones entering into the composition of the right half of the skull and face, the abnormal condition of the articulation of the lower jaw on that side being merely a consequence of the imperfect development of the bones composing it, and proper growth of the left half of the lower jaw. The case, however, is curious and interesting. Mr. Smith next describes the symptoms of the ordinary dislocation of the lower jaw, the detection of which is unattended with difficulty. When the luxation is confined to one side, it is not so easily recognised. The symptom which Mr. Hey found to be the best guide, in this case, is a small hollow which can be felt behind the condyle that is dislocated, which does not subsist on the sound side.

Mr. Smith notices the distortion of the countenance produced by chronic rheumatic arthritis of the temporo-maxillary articulation. "In the majority of instances, this remarkable disease attacks those of advanced age, and is symmetrical; but occasionally it occurs during the period of adult life. In the latter case, it is generally more rapid in its progress, is accompanied by greater pain, and is more liable to implicate the neck of the condyle and the ramus of the jaw." The nature of the distortion which attends this remarkable affection, varies according as one or both articulations are engaged in the disease.

"When it is confined to that of one side, and has existed for a considerable period, the mouth becomes crooked, the affected side of the jaw being drawn forwards and towards the opposite side, so that the teeth of the lower jaw, upon the sound side, project beyond those of the superior maxilla; but when the disease is symmetrical, the entire of the lower jaw is drawn forwards, and the chin projects just as it does (although from a different cause) in the edentulous subject.

"The glenoid cavity, when the disease has been of long duration, becomes increased as to its circumference, this enlargement being accomplished at the expense of the horizontal and transverse roots of the zygoma, more especially of the latter, which in all cases is to a greater or less extent absorbed.

"It is upon the destruction of this transverse root, or articular eminence, that the distortion of the countenance depends, for when the removal by absorption of this process is, to a certain extent, accomplished, the external pterygoid muscle then draws the jaw forwards and to the opposite side, where but one articulation is diseased, and the muscles of each side act in displacing it directly forwards, when the destruction of the articular eminence is symmetrical." P. 292.

We have quoted our author's account of this rheumatic affection of the jaw, as it is one which we have not hitherto recognised, and we believe will be new to most of our readers. In the last Chapter of this work,

Mr. Smith furnishes some additional observations on fractures of the radius, dislocations of the bones of the foot, and congenital luxation of the wrist-joint, in support of his views on these subjects.

Our notice of Mr. Smith's work has been so full that our readers will be able to form some estimate of its value. He has endeavoured to throw light on injuries and affections of an obscure, complicated, or rare character, and to assist surgeons in discriminating more clearly than heretofore the various lesions implicating the principal articulations. In the execution of this task he has collected many important and interesting facts, which he has described clearly and discussed without bias, and with a desire to advance our knowledge of surgery. The work is enriched with a large number of wood-cuts, which were really necessary to enable the readers of it to comprehend the descriptions in the text. These figures are well executed, but there is a hardness about most of them, and a want of that finish which may be observed in the best woodcuts which illustrate the works that have recently issued from the London press.

Should this work reach a second edition, we advise Mr. Smith to give his numerous extracts from the French writers in translation, instead of in, or in addition to, the original. He may reply that this is unnecessary, as every well-educated medical man in the present day is able to read the French language; which is true enough; but we know that there are a great many members of our profession who unfortunately are not well educated, or who are unacquainted with any other modern language than their own, but who may nevertheless desire to consult Mr. Smith's book, and it is for their benefit that we make this suggestion.

